# Pure Ultrasonic Communication in an Endemic Bornean Frog

**DOI:** 10.1371/journal.pone.0005413

**Published:** 2009-04-29

**Authors:** Victoria S. Arch, T. Ulmar Grafe, Marcos Gridi-Papp, Peter M. Narins

**Affiliations:** 1 Department of Ecology and Evolutionary Biology, University of California, Los Angeles, Los Angeles, California, United States of America; 2 Department of Biology, University Brunei Darussalam, Jalan Tungku Link, Gadong, Brunei Darussalam; 3 Department of Physiological Science, University of California Los Angeles, Los Angeles, California, United States of America; University of Sussex, United Kingdom

## Abstract

*Huia cavitympanum*, an endemic Bornean frog, is the first amphibian species known to emit exclusively ultrasonic (i.e., >20 kHz) vocal signals. To test the hypothesis that these frogs use purely ultrasonic vocalizations for intraspecific communication, we performed playback experiments with male frogs in their natural calling sites. We found that the frogs respond with increased calling to broadcasts of conspecific calls containing only ultrasound. The field study was complemented by electrophysiological recordings from the auditory midbrain and by laser Doppler vibrometer measurements of the tympanic membrane's response to acoustic stimulation. These measurements revealed that the frog's auditory system is broadly tuned over high frequencies, with peak sensitivity occurring within the ultrasonic frequency range. Our results demonstrate that *H. cavitympanum* is the first non-mammalian vertebrate described to communicate with purely ultrasonic acoustic signals. These data suggest that further examination of the similarities and differences in the high-frequency/ultrasonic communication systems of *H. cavitympanum* and *Odorrana tormota*, an unrelated frog species that produces and detects ultrasound but does not emit exclusively ultrasonic calls, will afford new insights into the mechanisms underlying vertebrate high-frequency communication.

## Introduction

The nearly universal ability of mammals to hear high-frequency sounds has led this taxon to be considered uniquely specialized among vertebrates. Humans are among the poorer performers in the class; our putative high-frequency cut-off is 20 kHz, and we have labeled frequencies above this boundary “ultrasound”. This anthropocentric designation ignores the fact that the vast majority of mammals tested hear well into the ultrasonic range, with specialists, such as echolocators, hearing up to and beyond 100 kHz. Non-mammalian vertebrates are comparatively restricted in their high-frequency hearing sensitivity. Birds have an upper limit of 8–12 kHz [1], and amphibians, reptiles and fish are generally considered limited to 5 kHz [2], [but see 3], [4]. Given the discrepancy between the upper-frequency hearing ability of mammals and all other vertebrates, it has been argued that in the study of vertebrate high-frequency audition, hearing above 10 kHz should be considered noteworthy [5]. For the sake of generality, we adhere to the anthropocentric designation of extraordinarily high frequencies as those in the ultrasonic range, and consider “high frequencies” to be those exceeding 10 kHz.

Despite the prevalence of ultrasonic hearing in mammals, relatively few species are known to use these frequencies for intraspecific communication. This group includes microchiropteran bats [6], cetaceans [7], [8], and some rodents [9], all of which emit purely ultrasonic communication vocalizations. The small size of this group may reflect the inherent transmission limitations of high-frequency sounds [10]–[12], which reduces their utility as long-distance communication signals. Within certain environmental and behavioral contexts, however, ultrasonic communication may offer advantages, such as enhanced signal-to-noise ratio, avoidance of eavesdropping by predators or prey, and increased energetic efficiency (for discussion, see [13]), and thus be favored by selection.

The frog *Odorrana tormota* was recently shown to produce and detect ultrasounds [4]. Thus, *O. tormota* is the first non-mammal demonstrated to communicate ultrasonically. The ultrasonic communication system of *O. tormota* differs from those of mammals, however. Most notably, all of the frogs' calls have an audible dominant frequency (DF), typically from 5–7 kHz [14]–[17], rather than being purely ultrasonic signals. Peak auditory sensitivity of the male frogs corresponds approximately to the dominant energy of their calls, falling between 6–10 kHz (Yu Z-L, *unpublished data*, [4]). In addition to audible energy, *O. tormota* calls contain prominent harmonics that extend into the ultrasound [14]. The frogs' hearing range is similarly extended, reaching an upper sensitivity limit of 34 kHz [4]. Male frogs respond behaviorally when presented with playback of conspecific calls that are high-pass filtered to contain only the ultrasonic harmonics [4]. Therefore, the communication system of these frogs includes spectral energy spread over an extraordinarily wide bandwidth. Although *O. tormota* appear to rely principally on communication within the 5–10 kHz range, their sensitivity to higher-frequency call components may facilitate communication amidst dynamic, predominately low-frequency ambient noise produced by nearby streams and waterfalls [4], [14], [16].

Another Southeast Asian frog, the Bornean endemic *Huia cavitympanum*, was recently found to produce calls with substantial ultrasonic spectral energy [18]. Unlike *O. tormota*, however, the DF of *H. cavitympanum* calls is extremely variable, spanning from high frequencies to ultrasound. A subset of the species' calls contains only ultrasonic spectral energy [18], suggesting that *H. cavitympanum* may communicate with purely ultrasonic signals. If so, this would represent the first documentation of the independent evolution of this communication strategy in a non-mammalian vertebrate. Communicating effectively with calls containing exclusively high-frequency and/or ultrasonic energy may necessitate that the *H. cavitympanum* auditory system, unlike that of *O. tormota*, be tuned for optimal detection of these frequencies. If so, exploration of the physiological underpinnings of this sensitivity in an amphibian may help characterize fundamental mechanisms facilitating high-frequency detection by vertebrates.

*Huia cavitympanum*, to our knowledge, is one of only two non-mammalian vertebrates known to emit exclusively ultrasonic vocal signals, and the other, the blue-throated hummingbird (*Lapornis clemenciae*), does not appear to detect ultrasounds [19]. Our objective was to determine whether the frogs' ultrasonic calls are used for communication, or are an epiphenomenon of the vocal production system. To do so, we performed playback experiments with male frogs in their natural calling sites. In addition, we obtained electrophysiological recordings from the auditory midbrain, and laser Doppler vibrometer measurements of the tympanic membrane response to acoustic stimulation, to determine the sensitivity spectrum of the frogs' central and peripheral auditory systems.

## Materials and Methods

### Ethics statement

The experimental protocol adhered to the ABS Guidelines for the use of animals in research and was approved by the UCLA Animal Research Committee (Protocol # 094-086-51).

### Behavioral experiments

The field study took place from 6 to 13 July, 2008. Males of *Huia cavitympanum* were found calling on the steep banks of the Nyipa River near Camp 1, Gunung Mulu National Park, Sarawak, Malaysia (04°03′N; 114°51′E). Calling males were not abundant during the study period, and were typically separated by ≥5 m. The large inter-male spacing allowed relative confidence that the frogs' vocal behaviors were selectively affected by playback stimuli.

#### Acoustic stimuli

Ultrasonic (US) stimuli comprised eight exclusively ultrasonic calls recorded from four males of *H. cavitympanum* during the 2007 field season [18]. A set of audible (AUD) stimuli was also prepared by selecting eight calls from the pool of calls recorded in 2007 that had an audible (<20 kHz) dominant frequency (n = 411). The chosen calls were recorded with good signal-to-noise ratio, did not have abrupt amplitude modulation patterns that could produce transient broadband components, and were from the greatest number of frogs possible. In addition, the selected AUD calls had duration, DF, maximum frequency, minimum frequency and bandwidth within ±1 SD of the mean values of all the audible calls. We were unable to use the same standard when choosing calls for the US stimuli because of the comparative paucity of exclusively US calls recorded, and the desire to maximize the number of different males represented by the stimuli. Most of the US stimuli deviated from ±1 SD of the mean value of the pool of US calls in only one or two of the call parameters. The selected stimuli were resampled from 96 or 192 kHz to 500 kHz to minimize potential aliasing artifacts by the playback equipment, and their peak amplitudes were normalized using Audition 2.0 (Adobe). A portion of background noise adjacent to each call was then selected and used to synthesize background noise (BKG) files corresponding to each US and AUD stimulus. These files were used as negative controls for behavioral responses from the frogs to background noise and/or instrument artifacts during playback. The final US, AUD and BKG stimulus files were each 400 ms long.

#### Experimental set-up and protocol

Experiments were conducted on six nights during the brief window of *H. cavitympanum* male nightly calling activity, ∼18:00–21:00 h. Ambient temperature and humidity were measured nightly with a digital thermohygrometer (Traceable Humidity/Temperature Pen, Fisher Scientific) and ranged from 22.5–26°C and 89–100%, respectively. We attempted to assemble the playback and recording equipment at a constant distance from the focal male, producing minimal behavioral disturbance. Playback stimuli were delivered by Avisoft-SASLab Pro (Avisoft Bioacoustics, Version 4.4) from a PC laptop to the speaker (Avisoft Bioacoustics, Ultrasonic Speaker Magnat; freq. resp.: 1–55 kHz±7 dB) via a portable US playback interface with an integrated D/A converter (Avisoft Bioacoustics, UltraSoundGate Player 116). The playback system was battery powered. Vocalizations were recorded with a broadband microphone (G.R.A.S. 40 BE; freq. resp.: 0.2–97 kHz±2 dB) and preamplifier (G.R.A.S. 26 CB) and a portable digital recorder (Sound Devices 722) at 96 kHz sampling rate. The microphone was held adjacent to the speaker. The line-out of the playback device was connected to one of the input channels of the digital recorder, and the signal from the microphone was connected to another. Using two different channels enabled us to distinguish between the playback stimuli and the vocal responses of the frogs.

The playback experiments were organized in a block design containing five (n = 5) or seven (n = 2) blocks. Blocks were 3 minutes long and arranged in the following order: (i) No Stimulus (NS1): natural calling activity recorded without playback; (ii) one of the US or BKG files, randomly selected to control for order effects; (iii) No Stimulus (NS2); (iv) US or BKG stimulus not played during the second block; (v) No Stimulus (NS3). In two of the trials, we employed a seven block design by adding an AUD stimulus and an additional No Stimulus block (NS4). In these trials, the US stimulus was the second block and the AUD was either the fourth block (frog #29) or the sixth block (frog #26) (Table 1). The presentation of a full set of blocks constituted one trial. Each male was used in a single trial. Complete data sets were collected from seven frogs. US, AUD and BKG files were looped to play every 10 s, although during one trial the US stimulus was presented every second for the first minute of the US block (frog #10), and during another the US and BKG stimuli were presented every 5 s for the duration of the blocks (frog #17). When a trial was finished, recordings of the playback stimuli were made from the position of the frog to estimate the stimulus sound pressure levels experienced by the frog.

**Table 1:** Number of calls emitted and calling rate during playback trials.

Frog ID	Block						
	1	2	3	4	5	6	7
7	NS1	US	NS2	BKG	NS3	-	-
	*1*	*68*	*27*	*0*	*0*		
		***22.67***					
26	NS1	US	NS2	BKG	NS3	AUD	NS4
	*0*	*45*	*8*	*0*	*0*	*58*	*6*
		***15.00***					
29	NS1	US	NS2	AUD	NS3	BKG	NS4
	*0*	*74*	*0*	*53*	*19*	*0*	*0*
		***24.67***					
15	NS1	BKG	NS2	US	NS3	-	-
	*0*	*0*	*0*	*42*	*12*		
				***14.00***			
21	NS1	BKG	NS2	US	NS3	-	-
	*0*	*1*	*0*	*84*	*76*		
				***28.00***			
10	NS1	US	NS2	BKG	NS3	-	-
	*20*	*103*	*41*	*18*	*1*		
		***43.00/31.00*** *					
17	NS1	US	NS2	BKG	NS3	-	-
	*0*	*96*	*89*	*76*	*32*		
		***32.00*** **					

Trials are organized by stimulus presentation order and playback rate. Blocks (3 min each) are identified as No Stimulus (NS), Ultrasonic (US), Background noise (negative) control (BKG), and Audible (AUD). Italicized values are the number of calls emitted by the focal male during each block. Numbers in bold are calling rates (calls/min) during US stimulus presentation. For the first five frogs in the table, the US and BKG stimuli were presented once per 10 s.

* Calling rate during the first minute of the US block when the stimulus was presented once per second, and during the second 2 min when the stimulus was presented once per 10 s.

** Calling rate for the US stimulus presented once per 5 s.

Upon completion of the behavioral experiments, the frogs were captured for electrophysiological characterization of their hearing range and measurements of the frequency response of their eardrums.

#### Data analysis

Calls produced during each experimental block were visually counted in Audition 2.0, and the time during the block (*i.e.*, 0–180 s) that they were emitted was noted. Spectral and temporal parameters of each call including duration, minimum frequency, maximum frequency, bandwidth, DF and peak amplitude were measured interactively using SoundRuler [20]. Because *H. cavitympanum* calls are highly variable in amplitude envelope, the duration limits of a subset of the calls were determined manually.

### Electrophysiological recording

#### Animal preparation

*Huia cavitympanum* males (n = 6) were anesthetized by immersion in a 0.2% solution of tricaine methanesulphonate (MS-222; Sigma) and placed on crushed ice to minimize bleeding during surgery. The surgical site was coated with a very thin layer of topical anesthetic (Benzocaine, 7.5%; Del Pharmaceuticals, Inc.), then the skull over the dorsal surface of the mid-brain was exposed by incising and retracting the skin. A small circular hole was made in the skull overlying the optic tectum using a dental drill. The meninges were carefully removed over the electrode penetration site using fine surgical forceps and a minute hook. When surgery was complete, the exposed area was covered with a small piece of absorbent tissue (Kimwipe) and the skin was repositioned. The frogs were then wrapped in moist gauze to prevent dehydration and allowed to recover fully from anesthesia. Upon recovery, the frogs were immobilized with an intramuscular injection of d-tubocurarine chloride (5 µg/g body weight; Sigma) and covered again with moist gauze to facilitate cutaneous respiration. Because closure of the Eustachian tubes can influence response properties of the peripheral auditory system [21], the frogs' mouths were carefully checked to ensure that the Eustachian tubes were patent and not blocked by the floor of the mouth. The frogs were positioned for recording on a small piece of stiff foam, and placed on a vibration-isolated table (Newport, Model VH3048W-OPT) inside an anechoic sound isolation chamber (Industrial Acoustics Company, Inc.).

#### Stimulus presentation and calibration

Acoustic stimuli were delivered by BrainWare [Tucker Davis Technologies (TDT)] at 200 kHz sampling rate (TDT RP2.1). Stimuli consisted of tone bursts (50 ms duration, 5 ms rise/fall times, 1 stimulus/s) spanning 2–∼40 kHz (the upper limit of stimulus frequency differed ±3 kHz between frogs) with a 1 kHz step size.

Prior to beginning electrophysiological recordings, a microphone (G.R.A.S. 40 BE) was placed in the position of the frog's right tympanic membrane (TM) and the sound pressure generated by the stimulation system was calibrated from 2–50 kHz using VibroToolbox 0.9.1b (http://vibrotoolbox.sf.net). To perform this calibration, the software iteratively altered the amplitude of the stimulus tones emitted from an electrostatic speaker (TDT ES-1; freq. resp.: 1–95 kHz] until the microphone recorded a flat spectrum (within ±1 dB). Tones were calibrated to reach the position of the frogs' TM at the behaviorally relevant sound levels of 80 (n = 2) or 90 (n = 4) dB SPL (re 20 µPa rms). The calibrated output amplitudes were then transferred to BrainWare.

#### Electrophysiological recording

Using tungsten microelectrodes (2–4 MΩ impedance; FHC Inc.), auditory evoked potentials (AEPs) were recorded extracellularly from the *torus semicircularis* (TS), which is the primary midbrain auditory processing center of the anuran central nervous system. The microelectrodes were placed over the dorsal surface of the optic tectum and inserted using a remotely controlled microdrive (Marzhauser Wetzlar, PM10). Neural responses from the brain were bandpass filtered (pass band: 10–500 Hz) amplified 10,000× (A–M Systems, Model 1800), monitored visually (Brüel & Kjaer Precision oscilloscope, Model 2160A), and extracted using BrainWare. A white-noise search stimulus was broadcast while the electrode was advanced into the TS. When the TS was reached, each stimulus frequency was presented 20 times beginning with 2 kHz and ending 2–3 stimulus frequencies higher than the frequency where AEPs were no longer visible. The electrode was then advanced ∼150–200 µm to a new position within the TS (3–5 recording positions per frog). Data were imported into Matlab (MathWorks, release 14.3) for analysis with a custom-written script. The 20 recordings from each stimulus frequency and electrode depth were averaged, and then this average was low-pass filtered (100× decimation) to remove recording noise. Peak-to-peak amplitude (the difference in µV between the first negative and positive peaks) and latency (time in ms between stimulus onset and the first negative peak) of the AEP waveforms were calculated from the resulting signals.

When recording sessions were complete, the frogs were immediately re-anesthetized by immersion in MS-222 (0.3%) for measurements of their tympanic membrane vibration spectra.

### Tympanic membrane vibration

The mouth and Eustachian tubes of the anesthetized animal were checked to ensure they were clear of fluids. The frog was then placed on a foam base that supported the body and the nose in a natural position while leaving the bottom of the mouth freely suspended. A reference microphone (G.R.A.S. 40BE) was positioned 1 cm above the TM. Acoustic stimuli were broadcast from the loudspeaker (TDT, ES-1) placed 10 cm from the reference microphone. The vibration velocity spectrum of the TM in response to the calibrated acoustic stimulus was measured with a scanning laser Doppler vibrometer (Polytec, Germany, PSV-300) aimed at the center of the TM, at an angle normal to the plane of the membrane.

#### Calibration and recording

The playback stimulus was a 160-ms long frequency-modulated chirp rising at constant rate from 1.8–40 kHz. The output stimulus and the signals from the microphone and vibrometer were digitized at 102 kHz (Polytec PSV-Z-040-H). Input spectra were obtained by calculating the fast Fourier transformation (FFT) of the average of 10 sequential repeats with 6.25 Hz frequency resolution. An FFT calculated over a signal that is frequency modulated at a constant rate will exhibit amplitude values that are reduced by a constant factor. This factor was determined by calculating the FFT over a stimulus with the same FM slope, but with constant known amplitude. FFT amplitude values of the experimental recordings were then corrected accordingly. Stimuli were calibrated to reach the frog's TM at 80 dB SPL (±1 dB) using VibroToolbox (see Electrophysiological recording). In one trial, the ES-1 speaker was replaced by a conventional loudspeaker (Versa-Tronics DOB100R, driven by a NAD-3020A amplifier) placed 40 cm from the frog's TM and used to present stimuli from 0.2–1.8 kHz.

## Results

### Call characteristics

The calls of *H. cavitympanum* are highly variable within and among individuals. The majority of calls contain some degree of downward frequency modulation, which varies in timing, slope, bandwidth and degree of warble. The DF of the calls occurs nearly always (∼95% of the time) in the first harmonic (F0), and can vary over >15 kHz within individual repertoires. The F0 of calls with ultrasonic DF can contain audible frequencies if the FM descends into the audible range, or can be exclusively ultrasonic [18].

### Playback experiments

Evoked vocal responses (EVRs) of males of *H. cavitympanum* to the playback of US stimuli were robust and began almost immediately after onset of the first stimulus (9.29±4.34 s, mean±SD; n = 7). The EVRs included both audible and purely ultrasonic calls. Playback stimulus intensity at the position of the frog was 84.3±4.3 dB SPL (mean±SD; n = 17; if the frog moved during playback, the stimulus intensity was measured from each position in which the frog came to rest). Calling rate during the US stimulus blocks was 21±6 calls/min compared with a baseline rate of 0±0.15 calls/min during NS1 and BKG blocks. Call rates were also elevated during the NS control following the US block (*i.e.*, either NS2 or NS3 depending on the order of stimulus presentation) compared to the other control periods, presumably due to the post-stimulus excitatory state of the frog; call rates averaged 8±10 calls/min during these NS blocks. In the trial in which the US stimulus was broadcast at 1 stimulus/s during the first minute, the call rate during that minute was 43 calls/min, decreasing to 31 calls/min for the second 2 minutes of the block. When the US stimulus was broadcast at 1 stimulus/5 s, the calling rate was 32 calls/min (Table 1). These data suggest a correlation between US stimulus presentation rate and calling rate.

For the five trials in which the US and BKG stimuli were presented at the same rate (1 stimulus per 10 s), we compared the number of calls produced during the US stimulus blocks to those emitted during the BKG blocks and to those emitted during the NS blocks immediately preceding the US and BKG blocks. Because the NS1 and NS2 blocks could precede a US or a BKG block, we labeled them PreUS-NS and PreBKG-NS, respectively. A significantly greater number of calls was emitted during the US stimulus blocks (Friedman ANOVA: χ^2^ = 11.791, P = .008, n = 5) than the other blocks. On average, the number of calls produced during the PreBKG-NS period was higher than the BKG and PreUS-NS periods. This is most likely because the PreBKG-NS group includes NS blocks that followed US or AUD blocks, thus elevated calling activity continued into these NS periods due to the frogs' post-stimulus excitation. The number of calls emitted during the US stimulus periods is significantly higher than the average number produced during the PreBKG-NS blocks (Wilcoxon Signed Ranks, P = .043) (Table 2).

**Table 2:** Number of calls emitted in playback categories included in the statistical analysis.

Frog ID	PreUS-NS	US	PreBKG-NS	BKG
7	1	68	**27**	0
15	0	42	0	0
21	0	84	0	1
26	0	45	**8**	0
29	0	74	**19**	0
AVG	0.2	62.6	10.8	0.2
SD	0.45	18.38	11.95	0.45

PreUS-NS and PreBKG-NS are the No Stimulus blocks preceding the Ultrasonic and Background noise blocks, respectively. Bold values are the number of calls emitted during NS periods following US or AUD blocks; this calling activity may result from the post-stimulus excitatory state of the frog. The difference in the average number of calls produced during the blocks is statistically significant (P = .008), and the number of calls emitted during the US stimulus periods is significantly higher than the average number produced during the PreBKG-NS blocks (Wilcoxon Signed Ranks, P = .043).

**Figure 1:**
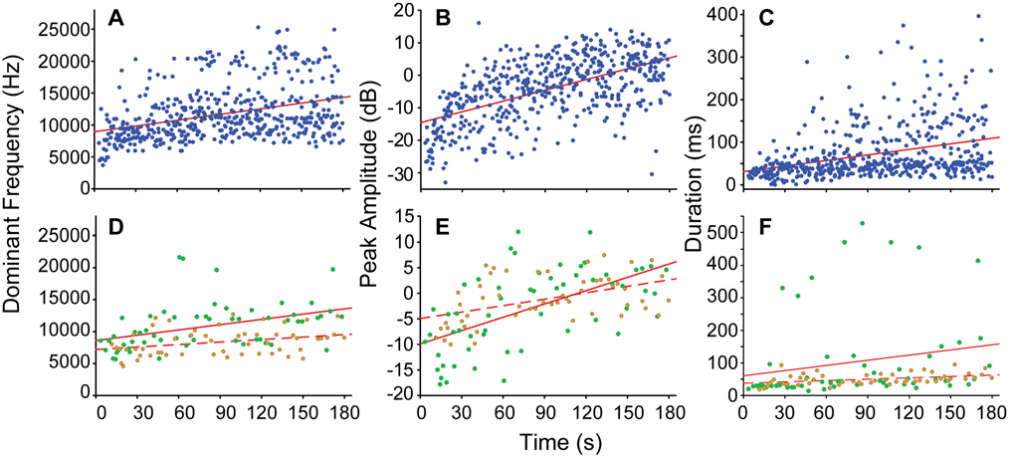
Change in evoked vocal response properties during ultrasonic and audible playback. Relationship between the (A) dominant frequency, (B) peak amplitude and (C) duration of evoked vocal responses to ultrasonic playback stimuli, and time of call emission during the 3-min playback period. Correlations are significant at the .01 level. D–F: A similar trend in call parameters is seen during the playback of audible stimuli to frog #26 (green circles, solid regression lines) and frog #29 (orange circles, dashed lines).

We also tested whether spectral and/or temporal characteristics of the frogs' EVRs to US stimuli changed over the course of the US playback blocks. For EVR DF and duration, we first removed the effect of individual by performing a univariate ANOVA (independent variable = frog; dependent variable = call DF or duration) and storing the residuals for use in further analyses. To evaluate changes in peak call amplitude during the US blocks, we calculated each frog's EVR peak amplitudes in dB relative to the frog's average EVR peak amplitude. We then performed Pearson correlation analyses to assess the relationship between (i) the DF and duration residuals, and amplitude dB values, and (ii) time of call emission during the US stimulus block (*i.e.*, 0–180 s). All three correlations were significant at the 0.01 level (Figure 1A–1C), with the average value of all three call parameters increasing with time of US stimulation.

Our primary experimental goal was to measure the frogs' behavioral response to purely US stimuli, but we also tested the effect of AUD stimuli on two frogs. The number of EVRs emitted during the US and AUD trials for these frogs was comparable (frog #26: 15 vs. 19; frog #29: 25 vs. 18) (Table 1). The DF, duration and amplitude of the EVRs produced during the AUD stimulus blocks showed a similar graded pattern of changes to that seen during the US stimulus blocks (Figure 1D–1F).

### Electrophysiology

**Figure 2:**
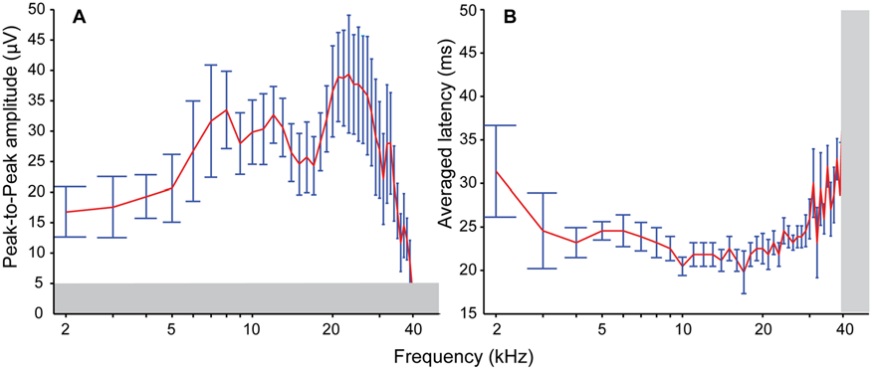
Average peak-to-peak amplitude and latency of AEP waveforms. (A) Peak-to-peak amplitude and (B) latency of the AEP waveforms (see text) recorded from the frogs' *torus semicircularis*. Error bars are ±1 standard error of the mean. Measurements from 2–34 kHz are from 6 frogs; from 35–37 kHz, n = 5; 38–39 kHz, n = 4; and 40 kHz, n = 3. The gray bars represent the noise floor of the recording system.

We measured AEPs from the TS of six males of *H. cavitympanum*. The averaged peak-to-peak amplitudes and latencies of the AEP waveforms evoked by response to tone bursts from 2–40 kHz (one penetration depth per frog) are shown in Figure 2A and 2B, respectively. AEP peak-to-peak amplitude is used as an index of auditory sensitivity, and the results from the *H. cavitympanum* TS show a broad range of high sensitivity spanning 6–33 kHz, with a slight dip between ∼14 kHz and 18 kHz. Sensitivity is maximal between approximately 20 kHz and 28 kHz. The averaged latency curve of the AEP waveforms approximates the species' audiogram [4], and further substantiates the wide frequency range of high sensitivity. Although sensitivity of the auditory midbrain decreases for frequencies greater than 30 kHz, AEPs were consistently observed up to 34 kHz, and were measurable up to 38 kHz in five of the males tested.

### Tympanic membrane vibration

**Figure 3:**
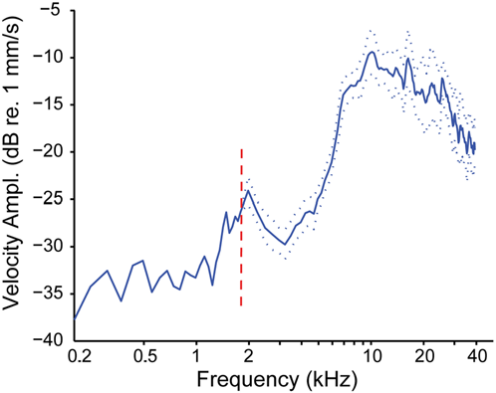
Velocity amplitude spectrum of *H. cavitympanum* eardrum movement in response to acoustic stimulation. From 1.8–40 kHz the average spectra of 11 measurements from 8 eardrums of 6 frogs is shown. Dotted lines denote the 95% confidence interval of the mean. Values below 1.8 kHz (delineated by the dashed vertical line), are from a single measurement.

The TM velocity amplitude spectrum of *H. cavitympanum* is shown in Figure 3. Between 1.8 kHz and 40 kHz, the mean curve was calculated from 11 trials performed on eight ears of six male frogs. Values below 1.8 kHz are from a single ear. These measurements reveal a wideband region of high sensitivity between approximately 7 kHz and 30 kHz. Above 30 kHz, the velocity amplitude drops off, matching the pattern of auditory midbrain sensitivity.

## Discussion

Playback of relevant acoustic stimuli has proven to be a reliable method of eliciting a behavioral response from anurans [*e.g.*], [ 22]–[28]. Accordingly, playback tests provide an excellent experimental tool by which natural and/or artificial stimuli can be manipulated to identify the functional elements of the focal species' acoustic signals. We presented *H. cavitympanum* males with a putative competitive stimulus comprised of the purely US call of a conspecific male. The frogs selectively vocalized in response to these stimuli (Table 1 and Table 2), providing behavioral evidence that they are sensitive to US frequencies, and that their exclusively US calls are relevant intraspecific and intrasexual signals. Males of *H. cavitympanum*, therefore, are the only non-mammalian vertebrates currently known to produce and detect vocalizations consisting of spectral energy entirely above the upper limit of human sensitivity.

Laser Doppler vibrometer measurements of the *H. cavitympanum* TM vibration velocity show that the middle ear of the species is sensitive over a wide range of frequencies, with a relatively flat plateau of maximal sensitivity spanning a broad high-frequency/ultrasonic range between 7 kHz and ∼30 kHz (Figure 3). Measurements from a single ear suggest that sensitivity of the peripheral auditory system decreases rapidly for frequencies below ∼2 kHz, although additional measurements are required to substantiate these data. Vibration velocity amplitude measurements of the stapes footplate (the structure that abuts the oval window at the junction between the middle and inner ear) of other frogs closely match TM data, suggesting that the high-frequency response measured at the eardrum of *H. cavitympanum* is most likely maintained across its middle ear [21], [29].

The TM velocity amplitude spectrum of the other frog that communicates ultrasonically, *O. tormota*, has been shown to depend on the state of the animal's Eustachian tubes (ETs, [21]). When the ETs are open, the resting condition, the peak sensitivity of TM vibration is *ca.* 7 kHz, closely matching the dominant frequency of the majority of the species' calls [14]–[17] and maximal sensitivity of their auditory midbrain (see below, Yu, Z. L. *unpublished data*, [4], [21]). With the ETs closed, which has been observed in the intact animal in the field during calling and swallowing, the TM velocity amplitude increases at high frequencies (10–32 kHz), showing a broad sensitivity plateau between ∼15 kHz and 25 kHz [21]. Preliminary observations of *H. cavitympanum* indicate that they lack morphological adaptations for ET closure (Gridi-Papp, *personal observation*, [21]). These data suggest an intriguing difference between the peripheral auditory tuning of *O. tormota* and *H. cavitympanum*. In the more common anatomical state (ETs open), *O. tormota* is most sensitive to frequencies between ∼5 kHz and 10 kHz, although their TMs and auditory midbrain retain some sensitivity to high frequencies extending into US. However, through the active closure of their ETs, the frogs can enhance their sensitivity to high frequencies. In contrast, the ETs of *H. cavitympanum* seem to be permanently open, and the vibration spectrum of their TMs suggests peak sensitivity to high frequencies. Thus, males of *H. cavitympanum* appear to be comparatively specialized for optimal perception of high frequencies, matching the predominance of these frequencies in their communication signals.

The same pattern holds for auditory evoked potentials (AEPs) recorded from the auditory midbrain of the two frog species. Peak-to-peak amplitude measurements of AEPs from the *H. cavitympanum* TS are greatest in the US range, between ∼20 kHz and 28 kHz (Figure 2A). The AEP latencies further substantiate a broad range of sensitivity to high frequencies (Figure 2B). In contrast, AEP measurements from the *O. tormota* TS show greatest sensitivity to frequencies below 5 kHz, falling off quickly in the US frequency range [4]. Although ET state was not monitored during the recordings from the *O. tormota* midbrain, the frogs were immobilized with a paralytic agent (d-tubocurarine chloride) that would presumably inhibit contraction of the submaxillary muscle that is primarily responsible for ET closure [21]; thus, the ETs were most likely open during these experiments. Given the effect of ET closure on the *O. tormota* TM sensitivity to US, we hypothesize that a similar increase in US sensitivity with ET closure would be seen in the downstream midbrain response. The electrophysiological data from *O. tormota* and *H. cavitympanum* corroborate that males of *H. cavitympanum* are more specialized for high-frequency hearing than those of *O. tormota*, with peripheral and central auditory systems tuned for maximal sensitivity to high frequencies.

The evidence that males of *H. cavitympanum* are specialized for high-frequency/ultrasonic detection gives rise to the question: what are the selection pressures that resulted in an upward shift of its communication channel? It is hypothesized that the inclusion of high frequencies in the *O. tormota* communication system resulted from the need to increase call audibility amidst a preponderance of broad-band, predominately low-frequency background noise [4], [14]. The *H. cavitympanum* population we have studied also lives alongside a rushing river, which produces similarly intense, broadband ambient noise [18]. Thus, pressure to increase the signal-to-noise ratio of intraspecific vocalizations could be a selective force fostering the convergence of these frog species on high-frequency communication.

Mammalian ultrasonic communicators can give us insight into additional selective pressures that may promote high-frequency communication specialization in *H. cavitympanum*. It has been postulated that the primary selective advantage of the broadly occurring high-frequency sensitivity in mammalia is that it enables the use of binaural spectral-difference cues to localize sound [5], [30]. These cues are strongest for frequencies with wavelengths short enough to be significantly blocked by an animal's head. The smaller the head, the higher these frequencies must be. Thus, small species can significantly increase their localization ability through detection of ultrasounds. Additionally, small animals, which comprise the majority of ultrasonic communicators (*e.g.*, rodents, bats), can attain substantial energetic advantages by using high frequencies/ultrasounds for communication, since the most efficient sound production occurs when the sound's wavelength matches the size of the vibrating structure [31]–[34]. Because frogs typically cannot detect high frequencies, they are unable to use these frequencies for communication; the resulting mismatch between the dimensions of their sound radiator (*i.e.*, vocal sac) and call frequencies is likely to be a primary cause of the extraordinarily low efficiency of frog sound production [31], [34]. Therefore, in addition to increased signal-to-noise ratio of calls, localization ability and energetic efficiency may be selective advantages encouraging high-frequency communication in anurans.

There is some preliminary indication that increased localization acuity and energetic benefits may be realized through use of high-frequency sounds by frogs. First, Shen et al. (2008) demonstrated the extraordinary localization ability of *O. tormota*, which rivals the best of the vertebrates [16]. The localization acuity of *H. cavitympanum* is unknown, but if males exhibit similar performance to *O. tormota*, it would provide correlational evidence that localization acuity is a selective advantage of high-frequency hearing in anurans. Accurate localization can aid animals to evade predators, obtain prey, and locate conspecifics with minimal energetic expenditure. Second, our behavioral data suggest that males of *H. cavitympanum* may accrue an energetic benefit through the use of high frequencies in their calls. During the agonistic encounters simulated by playback, focal males changed the properties of their vocal signals in a graded manner, significantly increasing call DF, duration and intensity (Figure 1A–1C). Increasing call duration and intensity is energetically expensive [31], [33]–[35] and has been hypothesized to indicate competitive ability and/or physiological condition of male frogs [36], [37]. Therefore, altering these call parameters potentially plays a role in the capacity to compete for females [38], [39]. *Huia cavitympanum* may compensate for the increased energetic demand of producing longer, louder calls by raising the calls' DFs, thus increasing the efficiency of coupling between the sound emitter and call wavelength.

Many intriguing questions remain to be explored in this system. For example, it is as yet unclear why the *H. cavitympanum* auditory system is maximally sensitive to US frequencies, given that exclusively US calls make up only a fraction of the species' vocalizations [18]. This heightened sensitivity may compensate for the more rapid attenuation of US components in their vocal repertoire, however further studies are required to substantiate this hypothesis. In addition, we do not yet know how the extraordinarily wide range of frequencies detected by *O. tormota* and *H. cavitympanum* is encoded by their two inner-ear auditory organs, the amphibian papilla (AP), and the putative high-frequency receptor, the basilar papilla (BP). If all frequencies contained within the *H. cavitympanum* calls are transduced by the BP, the relatively simple resonant structure and poor frequency resolution properties of this organ [40]–[42] may explain why the frogs showed no obvious behavioral discrimination between AUD and US playback blocks. Future studies will attempt to characterize the response to AUD playback more fully to determine conclusively whether the frogs behaviorally discriminate between US and AUD conspecific calls. Finally, the mating systems of these species are poorly understood. Selection on acoustic communication is likely to favor characteristics that increase the effective range of signaling in the context of mate attraction [34]. In the case of *H. cavitympanum*, the need to attract females with their advertisement vocalizations would seem to select against the nearly exclusive use of high frequencies/ultrasounds in their calls due to the vulnerability of short wavelengths to attenuation and scattering. It is possible that in this species gravid females vocalize to attract mates, as is the case in *O. tormota* [16]. If this is true, the spectral structure of the female calls may reflect the need for greater transmission distance and the male calls may be designed for short-range communication, which we emulated during our acoustic playback experiments.

*Odorrana tormota* and *H. cavitympanum* have apparently converged on the ability to use high frequencies and ultrasounds in communication. The data presented here make it clear that the mechanistic underpinnings of this ability differ in the two anurans. Most strikingly, *H. cavitympanum* can communicate with exclusively ultrasonic calls, similar to the vocalizations produced by mammalian ultrasonic communicators. Overall, our data suggest that *H. cavitympanum* is specialized for communication with high frequencies, while *O. tormota* increases its sensitivity to these frequencies by an active mechanical adjustment. Studies comparing the evolutionary, behavioral and physiological foundations of the high-frequency specialization of *H. cavitympanum* with the facultative induction of heightened high-frequency sensitivity in *O. tormota* will likely provide insight into the fundamental properties promoting high-frequency hearing in all vertebrates.
